# Synthesis, characterization, electrospinning and antibacterial studies on triphenylphosphine-dithiphosphonates Copper(I) and Silver(I) complexes

**DOI:** 10.1186/1752-153X-8-18

**Published:** 2014-03-14

**Authors:** Mehmet Karakus, Yuksel Ikiz, Halil Ibrahim Kaya, Omer Simsek

**Affiliations:** 1 Department of Chemistry, Faculty of Arts&Sciences, Pamukkale University, Kinikli 20075, Denizli, Turkey; 2 Department of Textile Engineering, Faculty of Engineering, Pamukkale University, Kinikli 20075, Denizli, Turkey; 3 Department of Food Engineering, Faculty of Engineering, Pamukkale University, Kinikli 20075, Denizli, Turkey

**Keywords:** Dithiophosphonates, Triphenylphosphine, Copper(I) and Silver(I) complexes, Nanofiber, Electrospinning, Antibacterial

## Abstract

**Background:**

The novel amido and O-ferrocenyldithiophosphonates [FcP(S)(SH)(NHR^1^)] (Fc = Fe(η^5^-C_5_H_5_)(η^5^-C_5_H_4_), R^1^ = 1-(4-fluorophenylethyl and benzyloxycyclopentyl) and [FcP(S)(OR^2^)S^−^][H_3_N^+^C(CH_3_)_3_] (R^2^ = myrtanyl) were synthesized by the reaction of [(FcPS_2_)]_2_ (Fc = Fe(η^5^-C_5_H_5_)(η^5^-C_5_H_4_)) and chiral amines, such as (S)–(−)-1-(4-fluorophenylethyl) amine and (1S,2S)-(+)-benzyloxycyclopentyl amine, and of (1S), (2S), (5S)-myrtanol in toluene. The reaction of ferrocenyldithiophosphonates and [Cu(PPh_3_)_2_]NO_3_ or AgNO_3_ and PPh_3_ gave rise to copper(I) and silver(I) complexes in THF. [Ag_2_{FcP(OMe)S_2_}_2_(PPh_3_)_2_] and [Cu(PPh_3_)_2_]NO_3_ were embedded into nanofibers and their antimicrobial activities on fibers were also investigated.

**Results:**

The compounds have been characterized by elemental analyses, IR, NMR (^1^H-, ^31^P-) spectroscopy as well as MS measurements. Nanofibers were obtained by electrospinning method which is the simplest and most effective method to produce nanoscale fibers under strong electrical field. Antimicrobial activity of the compound **5**, [Ag_2_{FcP(OMe)S_2_}_2_(PPh_3_)_2_], and [Cu(PPh_3_)_2_]NO_3_ on fibers were studied.

**Conclusions:**

In this study, the new dithiophosphonate ligands were synthesized and utilized in the preparation of copper(I) and silver(I) complexes with ferrocenyldithiophosphonate and triphenylphosphine. Then, the compounds [Ag_2_{FcP(OMe)S_2_}_2_(PPh_3_)_2_] and [Cu(PPh_3_)_2_]NO_3_ were added into the PAN solutions (Co-PAN dissolved in dimethylacetamide) and the solutions were electrospun onto microscope slides and PP meltblown surfaces. Antimicrobial activity of the compounds [Ag_2_{FcP(OMe)S_2_}_2_(PPh_3_)_2_] and [Cu(PPh_3_)_2_]NO_3_ on fibers were determined in vitro against two indicator strains; *M. luteus* NCIB and *E. coli* ATCC25922. The obtained results indicated that these metals showed moderate level antimicrobial activities.

## Introduction

Metallic silver and copper are natural antimicrobial agents and historically recognized [1,2]. These agents have been added into many polymer solutions, such as polyacrilonitrile (PAN), polyvinyl alcohol (PVA), Poly(N-vinylpyrrolidone), polylactic acid (PLA), to produce nanofibers with electrospinning method [3-10].

Electrospinning is a simple method to produce micro or nanoscale fibers. Nanofibers, due to their high surface area and porosity, find applications in energy storage, healthcare, biotechnology, environmental engineering, defense and especially filtration [11]. Electrospinning process uses a high voltage electric field to produce electrically charged jets from polymer solution. Polymer solution on tip of a syringe or pipette ejects toward opposite charged collector when overcome surface tension. Polymer chain entanglements prevent jets from breaking off and create fiber form. Because of evaporation and air drag, jets split into smaller diameters [12]. Process parameters are divided into; solution parameters which include viscosity, surface tension, electrical conductivity; processing conditions which include applied voltage, tip to collector distance, feeding amount and type; and ambient conditions which include temperature and moisture [13].

Dithiophosphonates are an important class in organophosphorus chemistry due to utilising in agricultural, medicinal and technological field [14-35]. It has been known that a considerable number of dithiophosphonates and their metal complexes have been easily synthesized by the reaction Lawesson’s reagent or Ferrocenyl Lawesson’s reagent and the respective alcohols or amines due to a ring opening reaction by nucleophilic attack [30-32]. However, there is no study on nanofibers of dithiophosphonates by using electrospinning method.

In the present work, we report the synthesis of novel dithiophosphonates and their metals complexes with dithiophosphonates and triphenylphosphine. All compounds were characterized by elemental analyses, IR, NMR (^1^H-, ^31^P-) spectroscopy as well as MS measurements. The compounds [Ag_2_{FcP(OMe)S_2_}_2_(PPh_3_)_2_] and [Cu(PPh_3_)_2_]NO_3_ added into PAN polymer solutions and mixed. Mixed polymer solutions were electrospun onto microscope slides and PP (polypropylene) meltblown surfaces. Meltblown is very commonly used textile nonwoven structure to support and protect fine fibers, especially in filtration. Antibacterial activities of those nanofibers were investigated.

## Result and discussion

### Synthesis and characterization

**Scheme 1:**
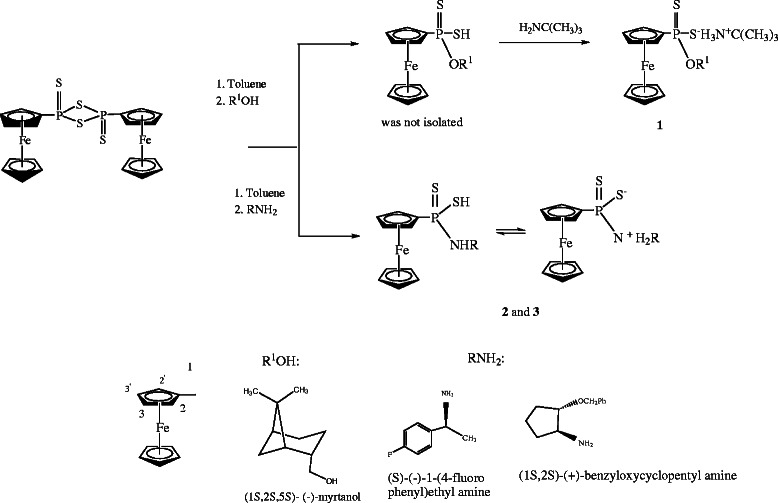
Synthesis of 1–3 and assigment scheme for ferrocene group.

**Scheme 2:**
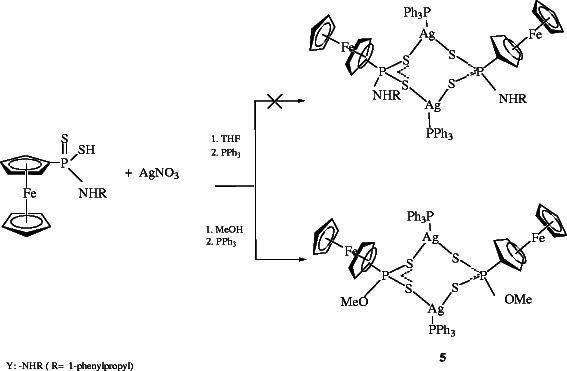
**Synthesis of [Ag**_**2**_**{FcP(OMe)S**_**2**_**}**_**2**_**(PPh**_**3**_**)**_**2**_**] 5.**

Amido and O-ferrocenyldithiophosphonates have been synthesized from Ferrocenyl Lawesson’s reagent and amines or (1S,2S,5S)- (−)- O-myrtanol (Scheme 1). The Ferrocenyl Lawesson’s reagent was reacted with (1S,2S,5S)- (−)- O-myrtanol and a crude dithiophosphonic acid was formed and then was treated with *tert*-butyl amine in order to convert it to its suitable salt **1**. In the case of amidodithiophoshonates **2** and **3** (Scheme 1), they were obtained as air stable solids [35]. The compound **5** was prepared by the reaction of (R) - (+) – 1 - phenylethyl amidoferrocenyldithiophosphonate [35] and AgNO_3_ in toluene and MeOH mixture (Scheme 2).

The IR spectrum of the ligands and their complexes showed two characteristic bands at around 692–642 cm^−1^ and 582 – 515 cm ^−1^ which are assigned to ν_as_(PS_2_) and ν_s_(PS_2_), respectively [36,37].

Mass spectra of the compound **1** – **5** exhibited m/z values for identifiable certain fragments. Specific rotations of all compounds showed that only one optical isomer was formed.

The ^31^P NMR spectra of the ligands **2** and **3** were measured in DMSO-d_6_ and showed two separate sets of signals which were shifted to 61.80 ppm (J_PN-H_ = 41.7 Hz for **2**) and 62.09 ppm (J_PN-H_ = 38.2 Hz for **3**) [35,38]. A very small signal was observed in the ^31^P NMR spectra of the ligands **2** and **3** due to probably neutral and zwitter ion form in the DMSO-d6 solution (see Scheme 1 for two isomer of **2** and **3**).

All ligands **1**–**3** reported here have been characterized by elemental analysis, IR, NMR and mass spectroscopy (Additional file 1). However, the ^31^C-NMR spectra of the ligands **2** and **3** did not measured due to decomposed in the DMSO-d_6_.

**Scheme 3:**
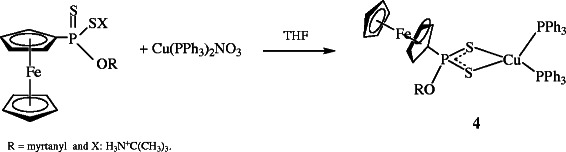
Synthesis of copper(I) complex 4.

The synthesis of copper(I) and silver(I) complexes with ferrocenyldithiophosphonate and triphenylphosphine have been described and also characterized by elemental analyses, IR, NMR and MS spectroscopies (Additional file 1). The synthesis of copper(I) complexes were performed by the reaction of [Cu(PPh_3_)_2_]NO_3_ and the ligands (Scheme 3).

The complex **4** was obtained as yellow-orange solid. The ^31^P NMR spectrum of **4** showed two signals at 97.8 and −2.9 ppm as expected [36] which were assigned to PS_2_ and PPh_3_, respectively. The Cu(I) and Ag(I) complexes of **2** and **3** also showed two signals in the ^31^P NMR spectrum as expected. However, other spectroscopic data were not satisfied. The ^31^P NMR spectra of [Ag_2_{FcP(OMe)S_2_}_2_(PPh_3_)_2_] **5** was measured in CDCl_3_ and observed two signal at 92.82 (PS_2_) and 6.03 (PPh_3_) ppm.

### Electrospinning studies

**Figure 1:**
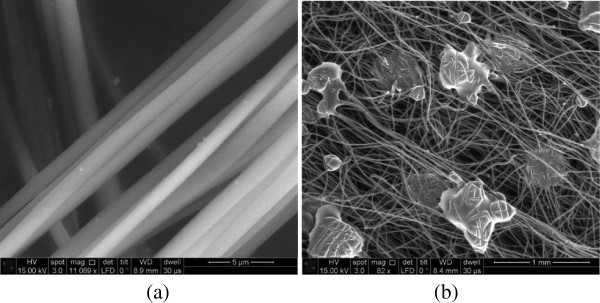
**[Ag**_**2**_**{FcP(OMe)S**_**2**_**}**_**2**_**(PPh**_**3**_**)**_**2**_**] added; a) electrospunned PAN fibers on glass, b) electrosprayed PAN particules on nonwoven surface.**

A comparative study on Silver(I) and Copper(I)- triphenylphosphine derivatives was performed and developed for the application of electrospun nanofibers. Figure 1 shows the compound [Ag_2_{FcP(OMe)S_2_}_2_(PPh_3_)_2_] added PAN nanofibers on a microscope slide and PP meltblown surface. Average fiber diameter on microscope slide was measured about 1 micron which was higher than expected average fiber diameter. Occasional electrospraying occurred as in Figure 1-b, because of aggregation of the compound [Ag_2_{FcP(OMe)S_2_}_2_(PPh_3_)_2_] particles.

**Figure 2:**
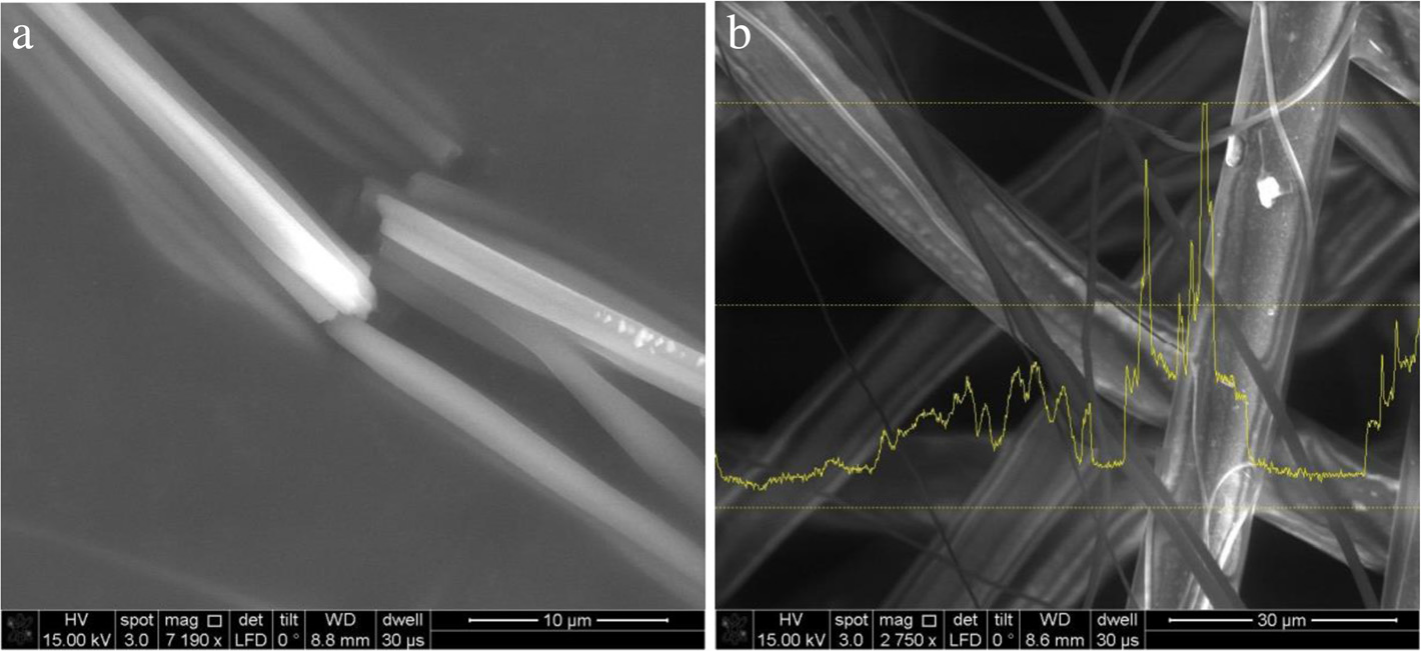
**[Cu(PPh**_**3**_**)**_**2**_**]NO**_**3 **_**added electrospunned PAN fibers; a) on glass, b) on nonwoven surface.**

Figure 2 shows [Cu(PPh_3_)_2_]NO_3_ added PAN nanofibers on a microscope slide and PP meltblown surface. Cu particles on nanofiber surface can be seen from SEM images as in Figure 2-a. Average PP meltblown fiber diameter was measured about 15 micron.

### Antibacterial activities

**Figure 3:**
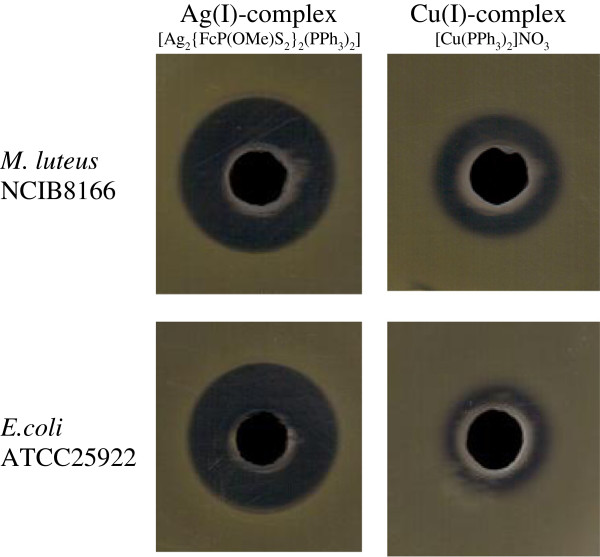
**Antimicrobial activity of [Ag**_**2**_**{FcP(OMe)S**_**2**_**}**_**2**_**(PPh**_**3**_**)**_**2**_**] and [Cu(PPh**_**3**_**)**_**2**_**]NO**_**3**_**.**

Antimicrobial activities of the compounds [Ag_2_{FcP(OMe)S_2_}_2_(PPh_3_)_2_] and [Cu(PPh_3_)_2_]NO_3_ were determined first on agar media against two indicator strains; *M. luteus* NCIBM and *E. coli* ATCC25922. According to the well diffusion assay on agar media, [Ag_2_{FcP(OMe)S_2_}_2_(PPh_3_)_2_] and [Cu(PPh_3_)_2_]NO_3_ showed medium level of antimicrobial activities against both strains (Figure 3). When the control compounds (not including Cu or Ag derivatives) were used for the same method, no inhibition zone or no antibacterial activity was occurred meaning that the relevant antimicrobial activities were mainly due to incorporated elements of Cu or Ag.

The control compounds and the compounds embedded fibers on meltblown surfaces were tested for inhibition of *E. coli* ATCC25922 in submerged bacterial solution. The highest inhibition (32.5 ± 2.1%) on *E. coli* was achieved with the compound [Ag_2_{FcPS_2_(OMe)}_2_(PPh_3_)_2_]. On the other hand, [Cu(PPh_3_)_2_]NO_3_ provided 19.4 ± 3.2% inhibition on *E. coli* while the control compounds showed no inhibition.

In this study, the compounds showed better antibacterial activities on agar media because of diffusion. However when the compounds embedded into fibers, they showed antibacterial activities only in contact with bacteria. Even though there was limited antibacterial activity, these metals could be used on fibers with dithiphosphonate and phosphine complexes for antibacterial applications.

It is generally believed that heavy metals react with proteins by combining the thiol (SH) groups, which leads to the inactivation of the proteins [39]. Therefore Ag and Cu could maintain their antimicrobial activity in the complexes of dithiphosphonate and phosphine. This is significant especially for using these metals as embedded in fibers, although they have limited antibacterial activity [40,41].

## Experimental

### Materials and method

Solvents were distilled before used. The compounds **4** and **5** were carried out under N_2_ atmosphere. All other chemicals were purchased from commercial sources and used directly without further purification. [FcPS_2_]_2_ (Fc: Fe(η^5^-C_5_H_5_)(η^5^-C_5_H_4_) and [Cu(PPh_3_)_2_]NO_3_ were prepared as described in the literature [32,42], respectively. Elemental analyses were determined with a GmbH varioMICRO CHNS apparatus. Melting points were determined by using Electrotermal apparatus. NMR spectra were recorded on a Bruker AVANCE DRX 400 NMR spectrometer and Jeol GSX 270 in CDCl_3_ and d_6_-DMSO. IR spectra was measured on a Perkin-Elmer 2000 FTIR spectrophotometer (4000 – 400 cm^−1^). Mass spectra were recorded with an AGILENT 1100 MSD and Waters machines. Optical rotation values were determined with an automatic digital ADP 440+ polarimeter.

### Electrospinning

**Figure 4:**
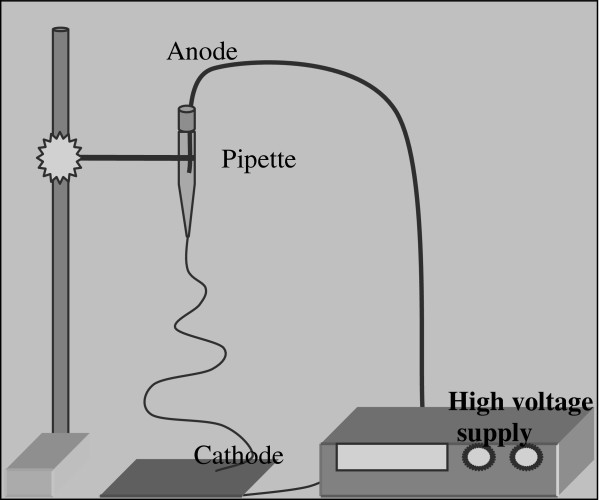
Electrospinning method.

The co-polymer polyacrylonitrile (PAN) and solvent dimethylacetamide (DMAc) were obtained from “AKSA acrylic chemistry company”. 15% polymer was dissolved in 85% solvent (w/w-weight by weight basis) at 80–100°C and stirred at least 4 hours. Polymer solution was prepared for electrospinning process by feeding into a pipette. Matsusada AU-40-0.75 high voltage supply were used to create electric field. Tip to collector distance was adjusted for 12 cm and voltage was adjusted 30 kV between the electrodes (Figure 4).

### Antibacterial activity

Two different antimicrobial test methods were used. Firstly the antimicrobial activity of synthesized compounds was determined by using well diffusion assay [43]. After filter sterilization of relevant compounds, approximately 100 μl was filled to the wells which had been prepared previously by overlaying LB soft agar including the indicator strains *Micrococcus luteus* NCBI8166 and *Escherichia coli*ATCC25922 on to the Müller-Hilton agar plates, then 5 mm wells were created with cork borer respectively. DSMO was used for controlling. To test antimicrobial efficiency of relevant compounds on fibers, the dynamic assessment of antimicrobial activity was carried out according to the standard test method released from American Society for Testing and Materials (ASTM) for immobilized antimicrobial agents under dynamic contact (E2149-01). Test bacteria (*Escherichia coli* ATCC25922) were cultured in LB broth (Fluka) overnight inoculations at 37°C. Subsequently, bacterial culture was diluted in 0.3 mM KH_2_PO_4_ buffer until the solution has an absorbance of 0.28 ± 0.02 at 475 nm as measured spectrophotometrically to reach bacterial suspension (1.5-3.0×10^5^ CFU ml^−1^). Rounds of fibers having total 4 in.^2^ treated surface area were inoculated with 50 ± 0.5 ml of bacterial suspension and incubated at 37°C 1 h ±5 min. Standard plate counts were performed after decimal dilution of the samples in 9 ml of 0.1% peptone water. The percent inhibition rate (%) was calculated as formula of (N1-N2/N1) × 100, where N1 and N2 represent the number of colonies on the plates before and after inhibition, respectively. Untreated fibers were used as a negative control.

### Synthesis of ^t-^Butyl ammonium salt of (1S,2S,5S)- (−)- O-myrtanyl ferrocenyl dithiophosphonate (1)

2,4-Bis(ferrocenyl)-1,3,2,4-dithiadiphosphetane-2,4-disulfide [FcPS(μ-S)]_2_ (1.80 g, 3.21 mmol) was reacted with 1S,2S,5S)-(−)-myrtanol (1.05 g, 6.42 mmol) in toluene (20 mL). The mixture was refluxed until all solids had dissolved. The dark brown solution was cooled to rt, filtered and treated with excess *tert*-butyl amine. The product was precipitated in freezer from toluene as a yellow solid, which was isolated by filtration, washed with toluene and *n-*hexane and then dried in air. Yield: 2.10 g 65%, m.p.: >187(dec.)°C. **[α]**_589_^25^ = −3.61 (c = 0.55 in THF). IR(KBr, cm^−1^) *ν*_max_: 648 (s,PS_2_, asym) and 582 (m, PS_2_, sym). ^1^H NMR (DMSO-d_6_, ppm) *δ*: 4.42 (br, 2H, C_5_H_4_), 4.23 (s, 5H, C_5_H_5_), 4.21 (br, 2H, C_5_H_5_), 4.18 (br, 2H, OCH_2_), 1.80-1.25 (m, 9H in myrtanyl group), 1.18 (s, 9H, *t*Bu), 1.02 (s, 3H, CH_3_), 1.01(s, 3H, CH_3_). ^13^C NMR (DMSO-d_6_, ppm) *δ*: 90.94 (d, C^1^; *ipso*-C in C_5_H_4_, ^1^*J*_P,C_ = 124.7 Hz), 84.23(d, ^2^J_P,C_ = 7.9 Hz), 71.30 (d, C^2^ and C^2′^, ^2^*J*_P,C_ = 13.9 Hz), 70.06 (s, C_5_H_5_), 69.71(d, ^4^*J*_P,C_ = 2.7 Hz), 68.91 (d, C^3^ and C^3′^, ^3^J_P,C_ = 4.9 Hz), 49.81 (s, *t*But), 49.12 (d, ^3^*J*_P,C_ = 5.2 Hz), 48.37, 41.50, 29.95 (s, *t*Bu), 29.71, 26.78, 26.41, 22.23, 20.8 ppm. ^31^P NMR (DMSO-d_6_ ppm) *δ*: 105.46. MS (ESI): m/z 433.1 [M–(H_3_N^+^C(CH_3_)_3_]. Anal. Calcd. for C_24_H_38_FeNOPS_2_: C, 56.80; H, 7.55; N, 2.76; S, 12.64%. Found: C, 57.08; H, 7.38; N, 2.72; S, 12.18%.

### Synthesis of (S) –(−)-1-(4-fluorophenylethyl)–amidoferrocenyldithiophoshonate (2)

[FcP(S)(μ-S)]_2_ 1.50 g (2.67 mmol) was treated with (S) – (−)-1-(4-fluorophenylethyl) amine (0.745 g, 5.35 mmol) in a 1:2 ratio in toluene (25 mL) to give the corresponding amidoferrocenyldithiophosphonate. The reaction was carefully heated until all the solids dissolved and a brown solution was obtained and then a solid product was formed, which was isolated by filtration. The product was washed with petroleum ether (40–60°C). The yellow crystalline product was dried under vacuum. Yield: 1.57 g, 70%, m.p.: 169°C. [α]_589_^25^ = 75 (c = 0.08 in THF). IR(KBr, cm^−1^) *ν*_max_: 645 (s, PS_2_, asym) and 526 (m, PS_2_, sym). ^1^H NMR (DMSO-d_6_ ppm) *δ*: 7.63 (br, 2H, arom.), 7.25 (br, 2H, arom.), 4.56 (br, 2H, C_5_H_4_), 4.43 (br, 2H, C_5_H_4_), 4.37 (s, 5H, C_5_H_5_), 2.50 (s, 3H, CH_3_), 1.59 (s, 1H, CH). ^31^P NMR (DMSO-d_6_ ppm) *δ*: 61.80 (d, J_PNH_ = 41.7 Hz) ppm. MS (ESI): m/z = 401.95 [M-F]^+^. Anal. Calcd. for C_18_H_19_NFPS_2_Fe: C, 51.56; H, 4.57; N, 3.34; S, 15.29%. Found: C, 51.71; H, 5.07; N, 3.54; S, 14.20%.

### Synthesis of (1S,2S)-(+)-benzyloxycyclopentyl–amidoferrocenyldithiophoshonate (3)

Compound **3** was prepared in the same manner as compound **2**, from [FcP(S)(μ-S)]_2_ (1.00 g, 1.78 mmol) and 1S,2S-(+)-benzyloxycyclopentyl amine 0.68 g (3.56 mmol) in toluene (25 mL). Yield: 1.19 g (76%), m.p.: 174–176°C. **[α]**_589_^25^ = 53.33 (c = 0.15 in THF). IR(KBr, cm^−1^) *ν*_max_: 645 (s, PS_2_, asym) and 525 (m, PS_2_, sym). ^1^H NMR (DMSO-d_6_ ppm) *δ*: 8.29(br, 1H, NH), 7.37(br, 5H, arom.), 4.54 (br, s, 2H, C_5_H_4_), 4.21 (br, s, 5H, C_5_H_5_), 4.18 (br, s, 2H, C_5_H_4_), 3.99 (br, 2H, OCH_2_), 3.80 – 1.69(br, m, 8H, C_5_H_8_ group). ^31^P NMR (DMSO-d_6_ ppm) *δ*: 62.09 ppm (J_PN-H_ = 38.2 Hz) ppm. MS (ESI): m/z = 296.86 [M-C_5_H_8_OCH_2_C_6_H_5_]^+^. Anal. Calcd. for C_22_H_27_NOPS_2_Fe: C, 56.06; H, 5.59; N, 2.97%. Found: C, 60.07; H, 6.34; N, 3.30%.

### Synthesis of [Cu{Fe(η^5^-C_5_H_5_)(η^5^-C_5_H_4_P(OR)S_2_)(PPh_3_)_2_}] (R = myrtanyl) (4)

A solution of [Cu(PPh_3_)_2_NO_3_] (0.13 g, 0.20 mmol) in THF(10 mL) was added dropwise to a solution of (1S, 2S, 5S)–O-myrtanyl-ferrocenyldithiophoshonate **1** (0.10 g, 0.20 mmol) in THF (10 mL) and stirred at r.t. for 2 h. A yellow-orange solution was observed. The reaction mixture was filtered and the solvent was removed under reduced pressure. A yellow-orange crystalline product was isolated. Yield: 0.12 g, 60%, m.p.: 179–180°C. **[α]**_589_^25^**=** 120 (c = 0.05 in THF). IR (KBr, cm^−1^) *ν*_max_: 642 (s, PS_2_, asym) and 515 (m, PS_2_, sym). ^1^H NMR (CDCl_3_, ppm) *δ*: 7.43 – 7.25 (m, 30H, arom.), 4.36 (br, 2H, C_5_H_4_), 4.25 (s, 2H, C_5_H_5_), 4.21 (s, 2H, C_5_H_4_), 3.80 (m, 2H, OCH2), 2.40–1.10 (m, 9H, in myrtanyl group), 1.24 (s, 3H,CH_3_), 0.87 (s, 3H, −CH_3_). ^31^P NMR (CDCl_3_, ppm) *δ*: 97.85 (PS_2_) and −2.87 (PPh_3_) ppm. Anal. Calcd. for C_56_H_56_OP_3_S_2_FeCu (1021.51 g.mol^−1^): C, 65.84; H, 5.52; S, 6.27%. Found: C, 65.49; H, 5.54; S, 5.93%.

### Synthesis of [Ag{Fe(η^5^-C_5_H_5_)(η^5^-C_5_H_4_P(OR)S_2_)(PPh_3_)_2_}]_2_ (R = CH_3_) (5)

A mixture of AgNO_3_ (0.12 g, 0.70 mmol) and PPh_3_ (0.18 g, 0.70 mmol) in MeOH (20 mL) was added dropwise to a solution of the compound (R) - (+) – 1 - Phenylethyl amidoferrocenyldithiophosphonate [35] (0.28 g, 0.70 mmol) in toluene (25 mL) and stirred for 2 h. A yellow precipitate product was immediately formed. The product was filtered, washed with petroleum ether(40–60°C) and dried in air. Yield: 0.38 g (79%). M.p.:>160°C(dec.). IR(KBr, cm^−1^) *ν*_max_: 649 (*ν*_asym_ PS_2_) and 560 (ν_sym_ PS_2_). ^1^H NMR (CDCl_3_, ppm) *δ*: 7.36 – 7.02 (m, 30H, arom.), 4.55 (br, 4H, 2× C_5_H_4_), 4.36 (br, 4H, 2xC_5_H_4_), 4.16 (s, br, 10H, 2xC_5_H_5_), 1.39 (d, br, 6H, 2xOCH_3_, ^3^J_P,H_ = 5.4 Hz). ^31^P NMR (CDCl_,_ ppm) *δ*: 97.82 (PS_2_) and 6.03 (PPh_3_). MS (ESI) (m/z): 279.1 [FcPS_2_]^+^. Anal. Calc. for C_58_H_54_O_2_P_4_S_4_Fe_2_Ag_2_: C, 51.12; H, 3.99; S, 9.41. Found: C, 50.76; H, 3.96; S, 9.87%.

## Conclusions

The new dithiophosphonate ligands were synthesized and utilized in the preparation of copper(I) and silver(I) complexes with ferrocenyldithiophosphonate and triphenylphosphine. Then, the compounds [Ag_2_{FcP(OMe)S_2_}_2_PPh_3_)_2_] and [Cu(PPh_3_)_2_NO_3_] were added into the PAN polymer solution (Co-PAN dissolved in dimethylacetamide) and the solution was electrospun onto microscope slide and PP meltblown surface producing fibers, average about 1 micron diameter. SEM images of these fibers show that compounds did not evenly distribute on fiber surface along the fiber length, meaning also not evenly distributed in polymer solution because of particles aggregation which caused electrospraying, as well. Antimicrobial activity of the compounds ([Ag_2_{FcPS_2_(OMe)}_2_(PPh_3_)_2_] and [Cu(PPh_3_)_2_]NO_3_) on fibers were determined in vitro against two indicator strains; *M. luteus* NCIB and *E. coli* ATCC25922. The obtained results indicated that these metals could be immobilized with the dithiophosphonate-phophine and showed moderate level antimicrobial activity.

## Competing interests

The authors declare that they have no competing interests.

## Authors’ contributions

MK has coordinated the experimental work, synthesized, characterized the structure of the all compounds and wrote the manuscript. YI has obtained nanofiber by electrospinning method. HIK and OS carried out antibacterial studies. All authors have read and approved the final manuscript.

## Supplementary Material

Additional file 1 Spectra of Compounds. Click here for file
