# Supplementary material for: Synthesis, characterization, electrospinning and antibacterial studies on triphenylphosphine-dithiphosphonates Copper(I) and Silver(I) complexes
Source: Chem Cent J. 2014 Mar 14;8:18. doi: 10.1186/1752-153X-8-18 (PMC4004045; doi:10.1186/1752-153X-8-18)
Supplement: Additional file 1 — Spectra of Compounds. [file 1752-153X-8-18-S1.doc]

**^t-^Butyl ammonium salt of (1S,2S,5S)- (-)- O-myrtanyl ferrocenyl dithiophosphonate (1)**

Fig.1 IR spectra of the compound 1

**
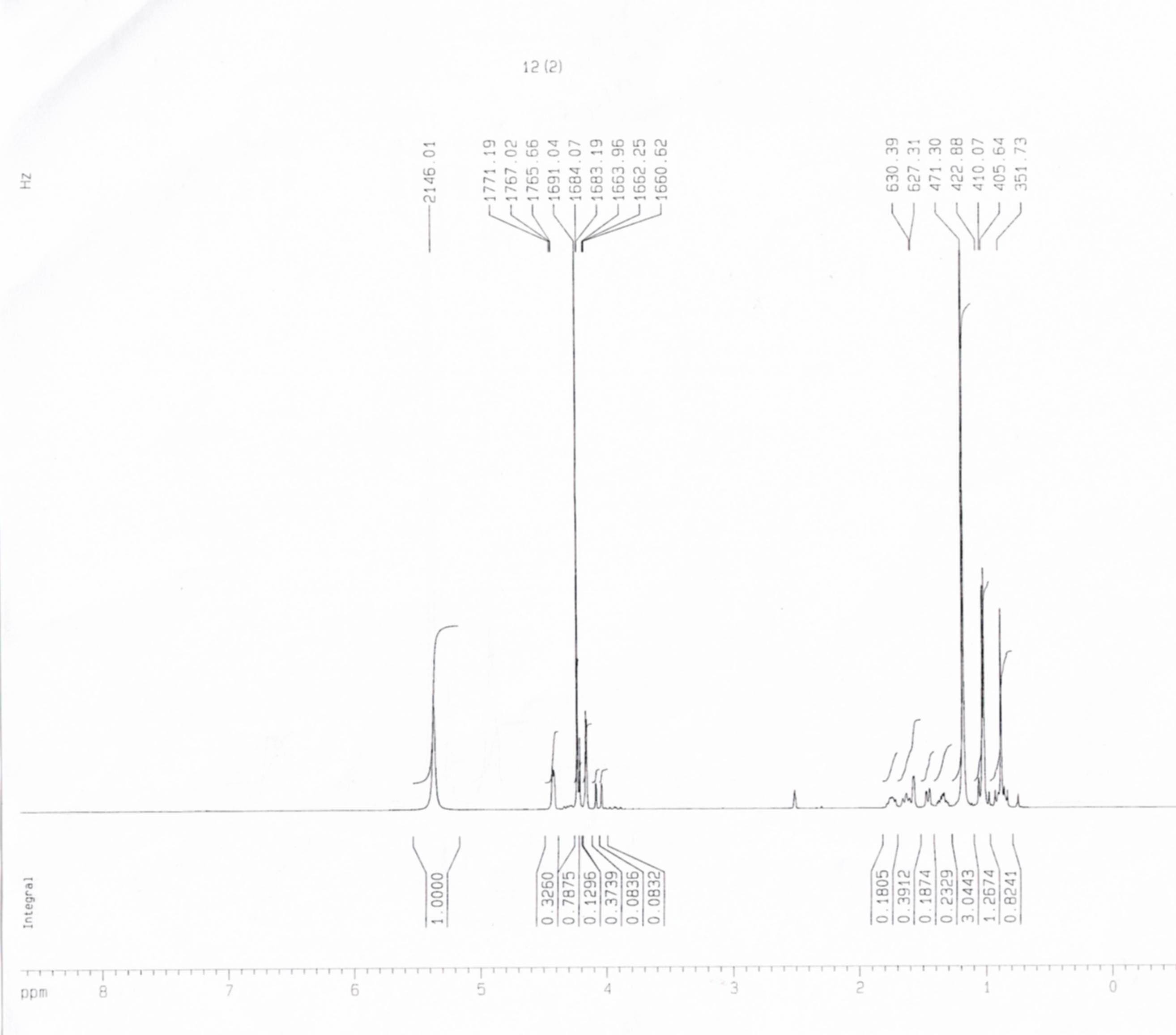
**

Fig.2. ^1^H-NMR spectra of the compound 1

**
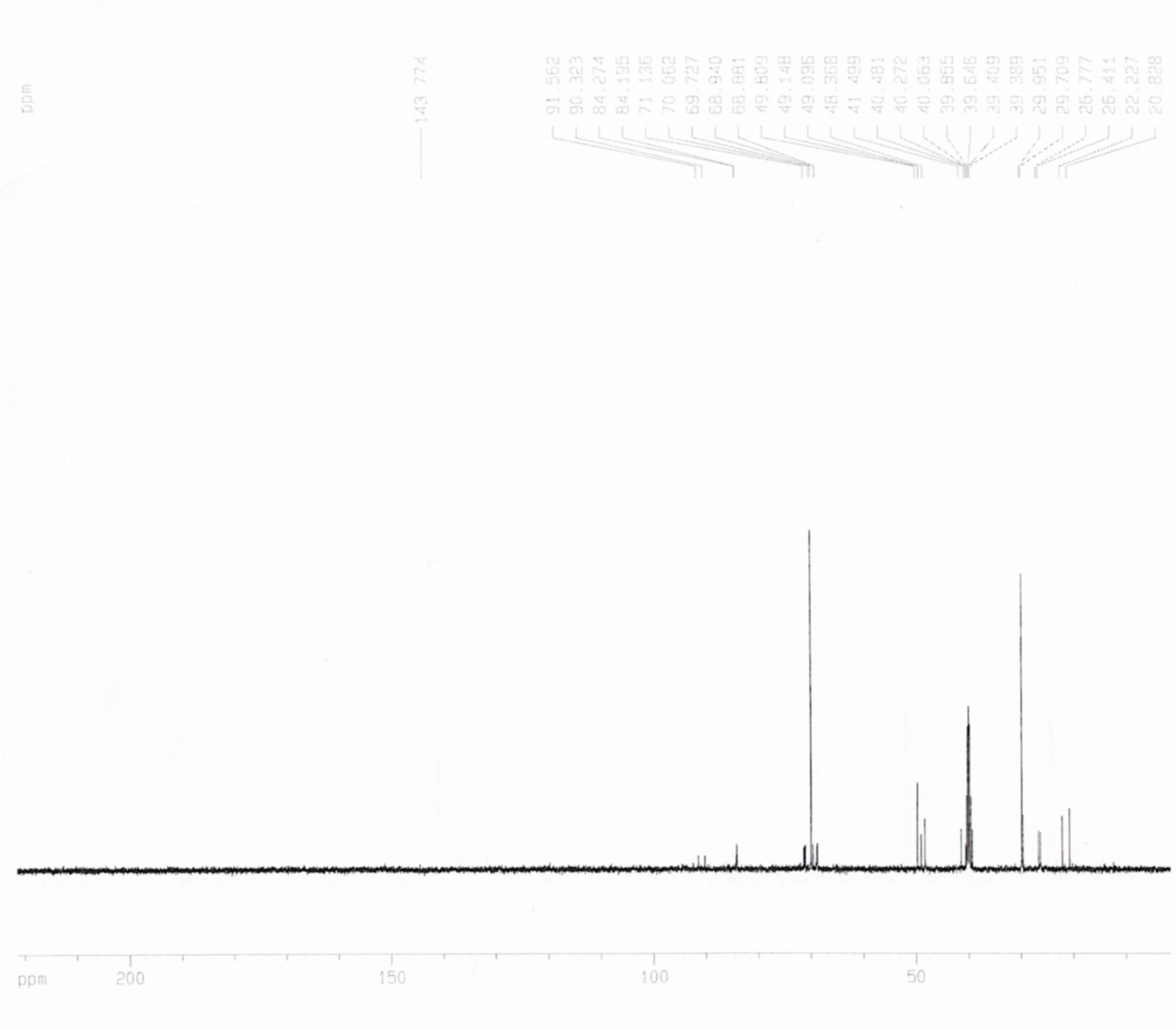
**

Fig. 3. ^13^C-NMR spectra of the compound 1


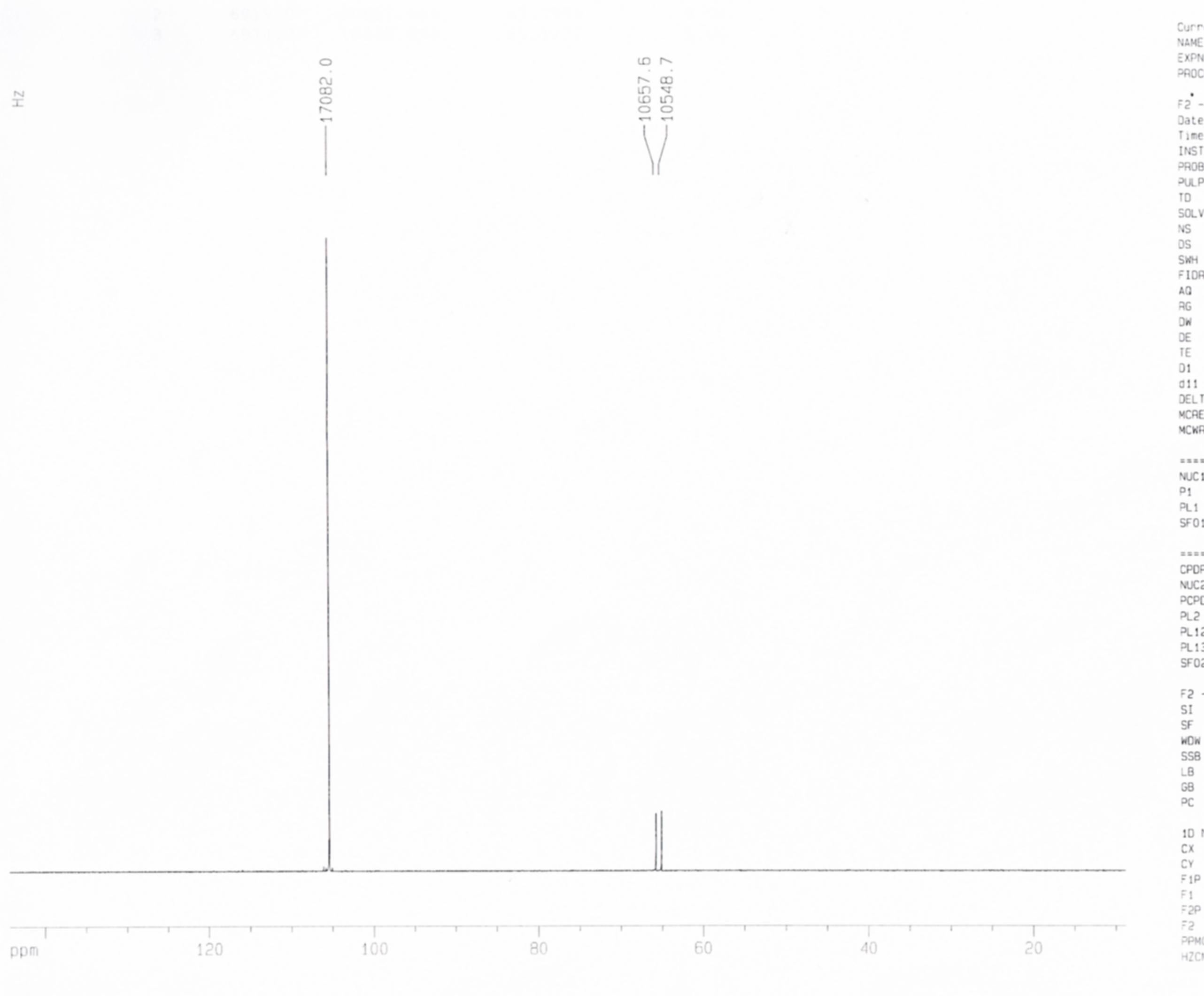


Fig.4. ^31^P-NMR spectra of the compound 1


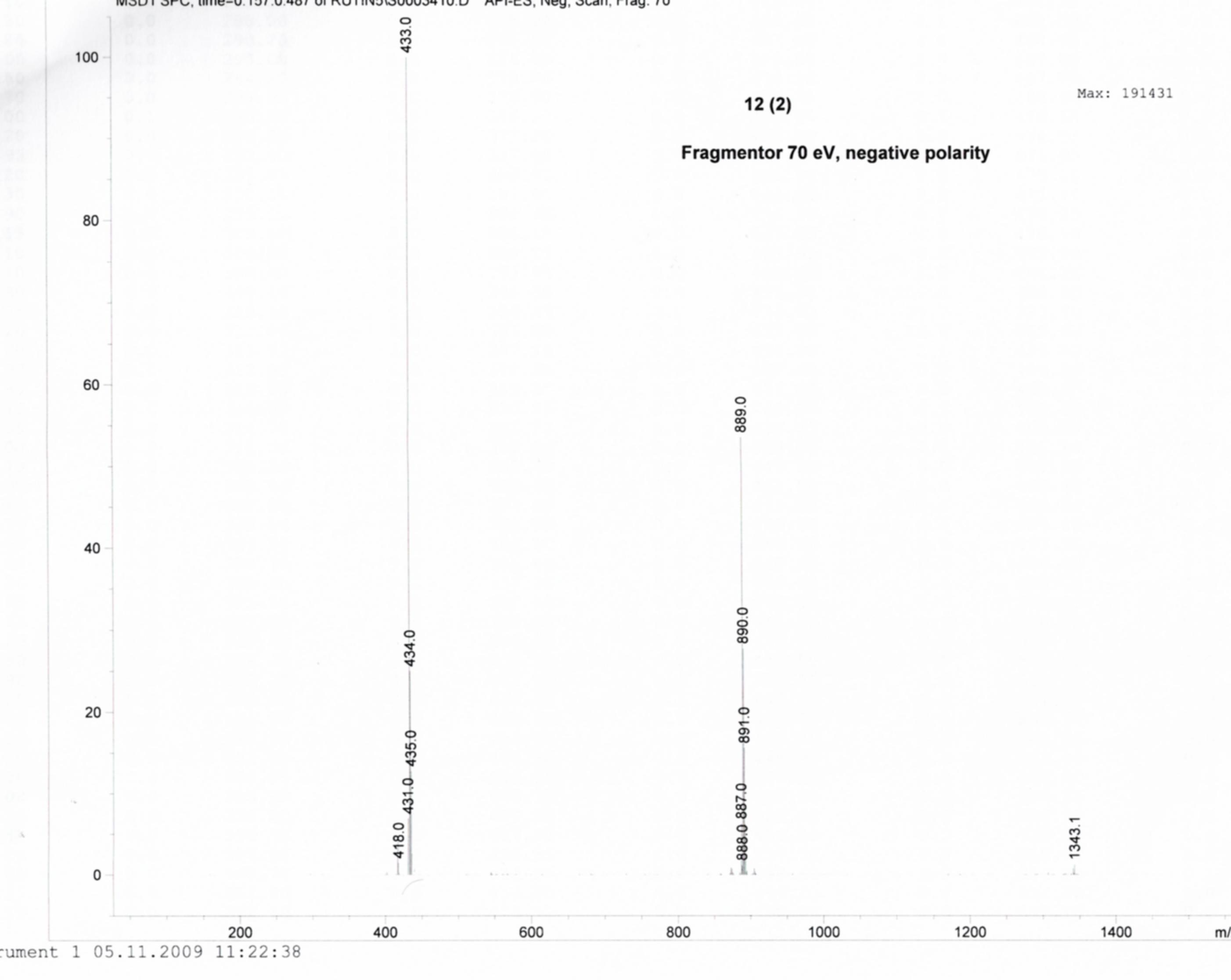


Fig. 5. Mass spectra of the compound 1

**(S) –(-)-1-(4-fluorophenylethyl)–amidoferrocenyldithiophoshonate (2)**

Fig.6 IR spectra of the compound 2


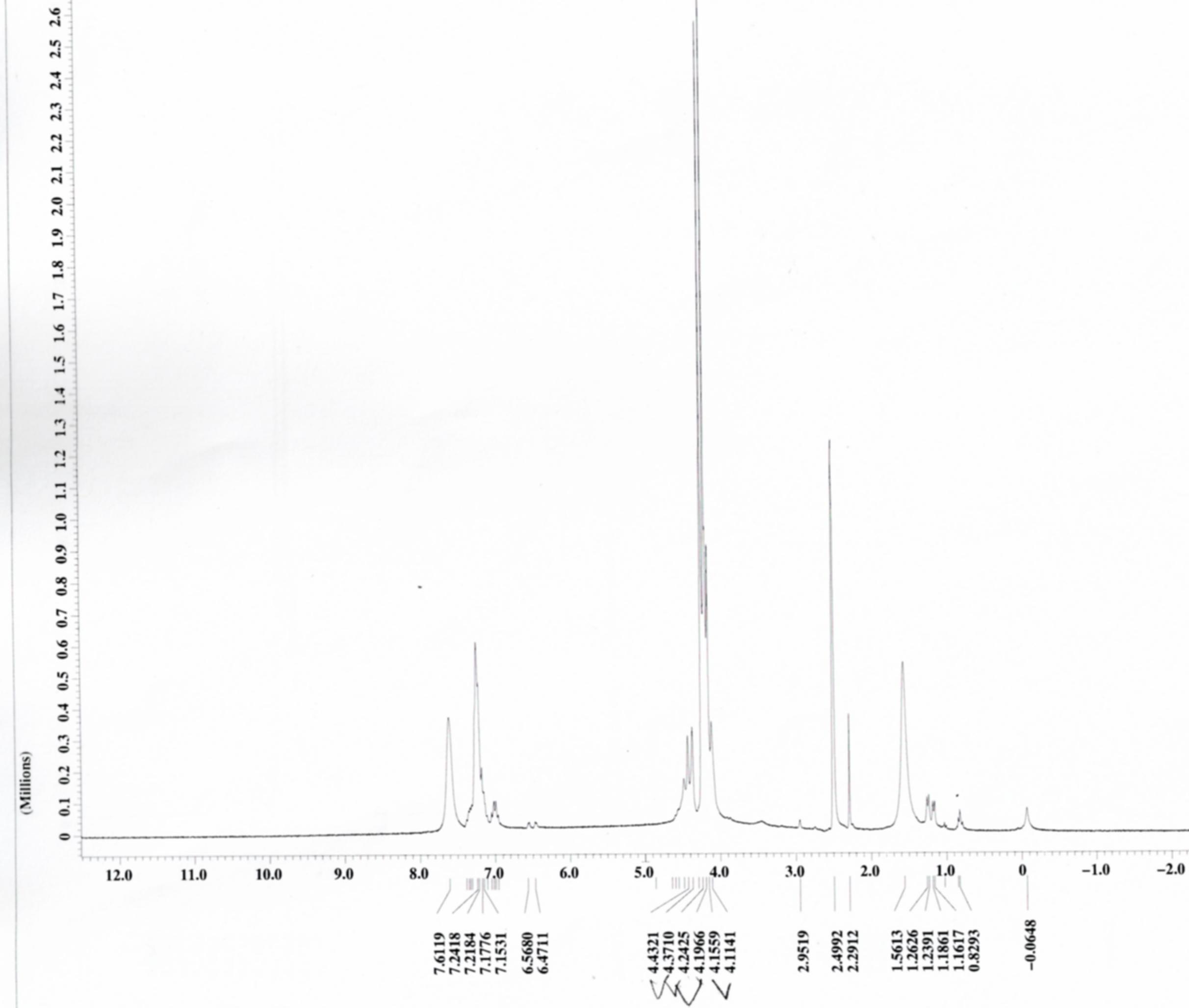


Fig.7. ^1^H-NMR spectra of the compound 2

**
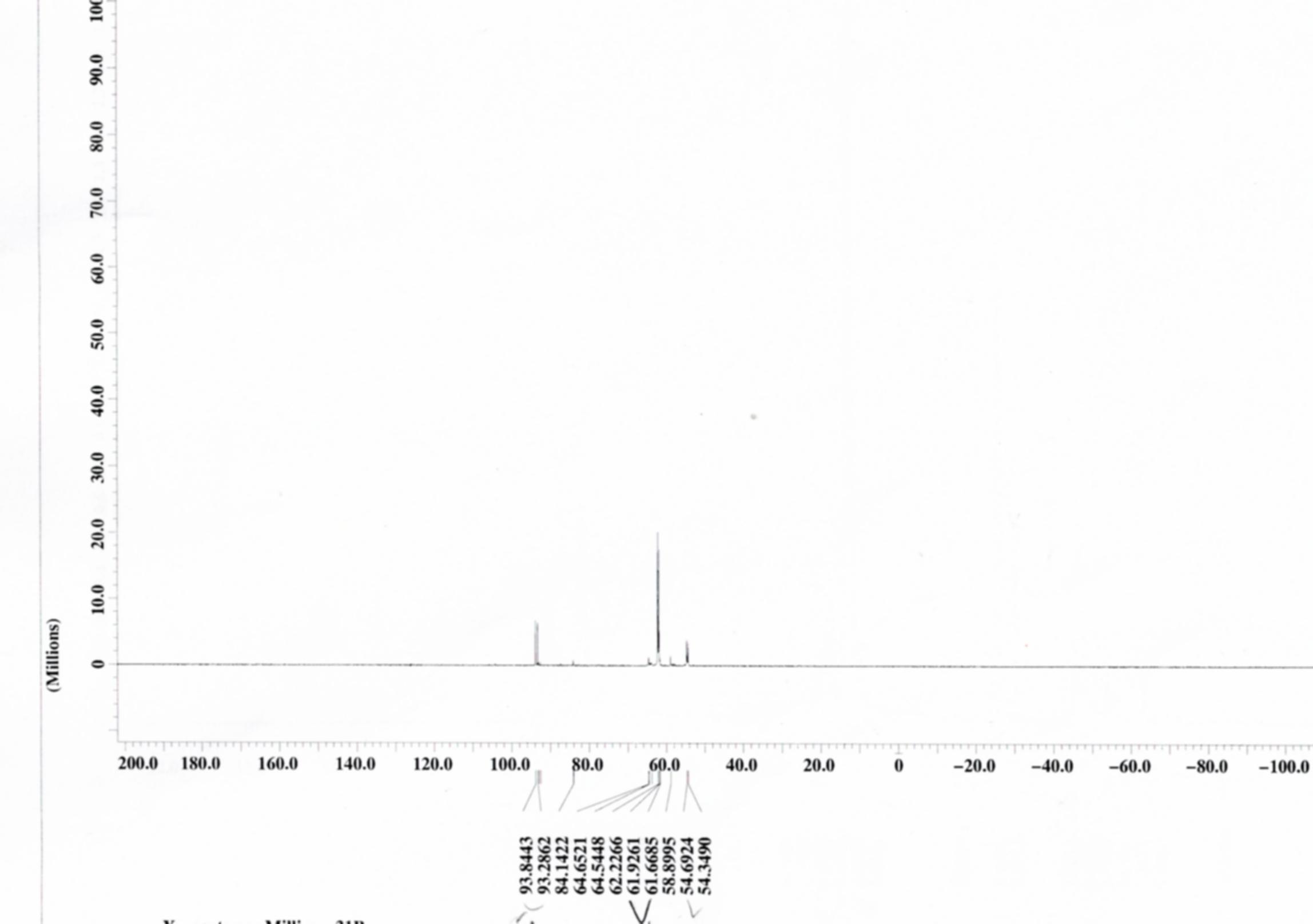
**

Fig. 8. ^31^P NMR spectra of the compound 2


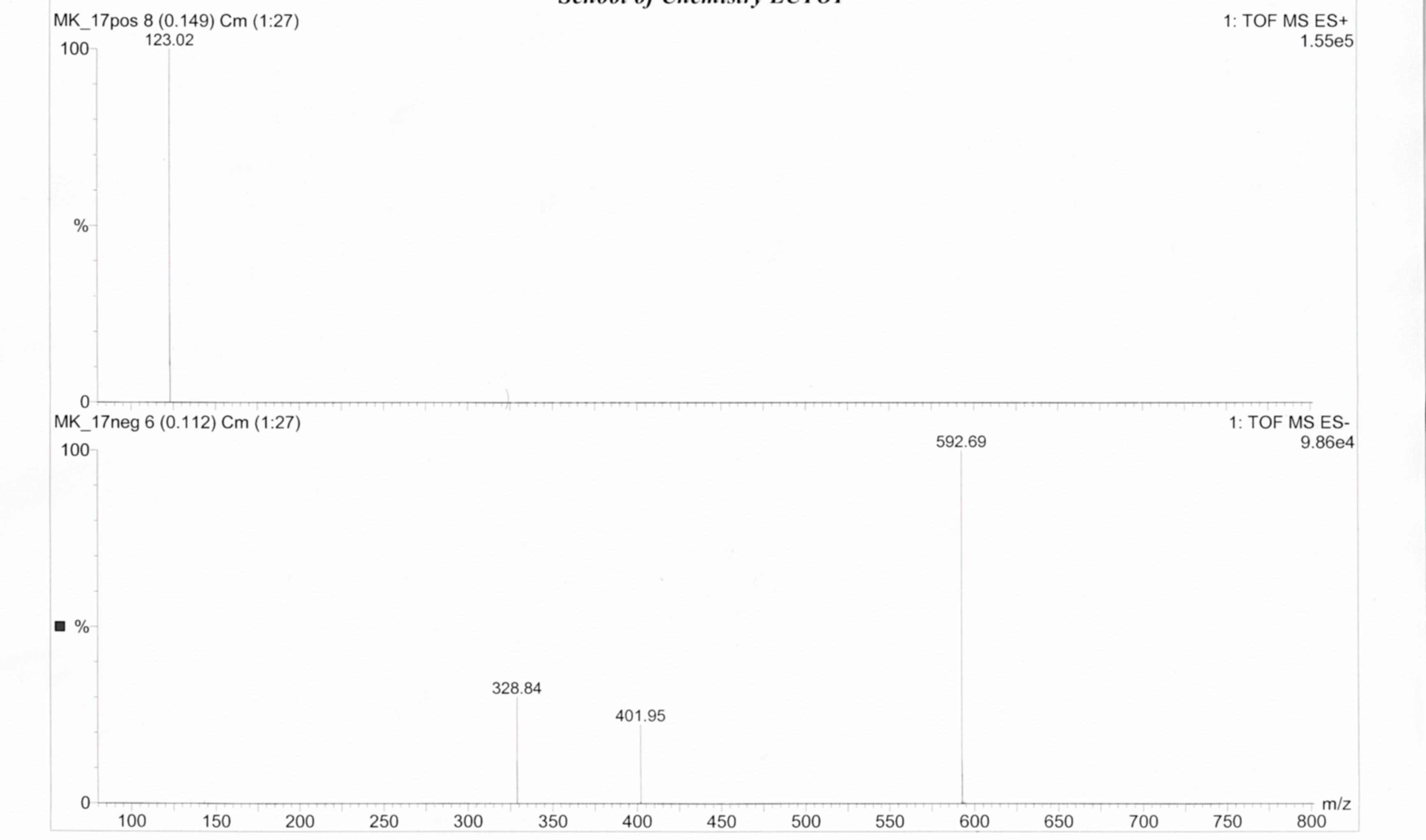


Fig. 9. Mass spectra of the compound 2

**(1S,2S)-(+)-Benzyloxycyclopentyl–amidoferrocenyldithiophoshonate** **(3)**

Fig.10. IR spectra of the compound 3


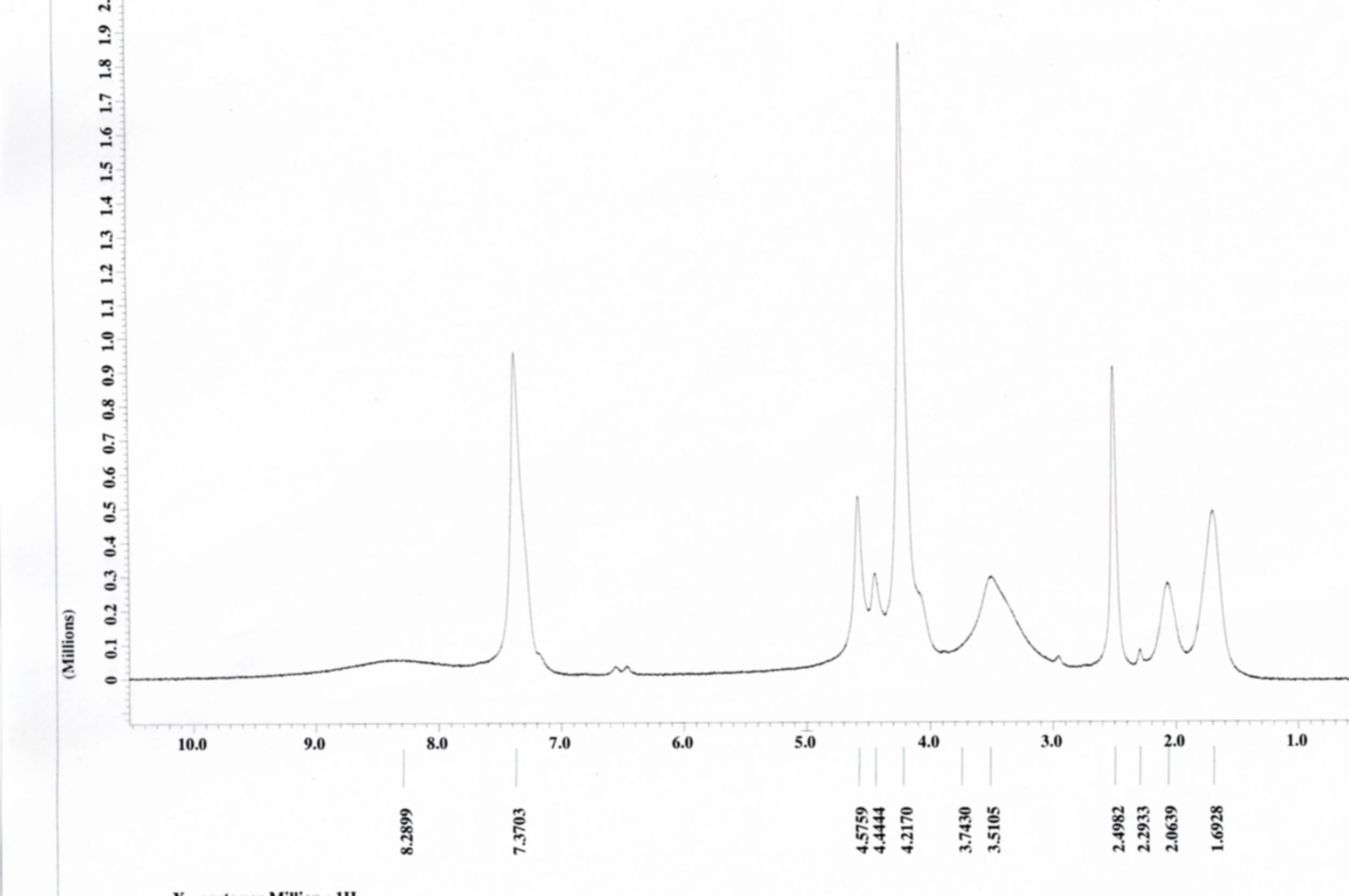


Fig.11. ^1^H-NMR spectra of the compound 3


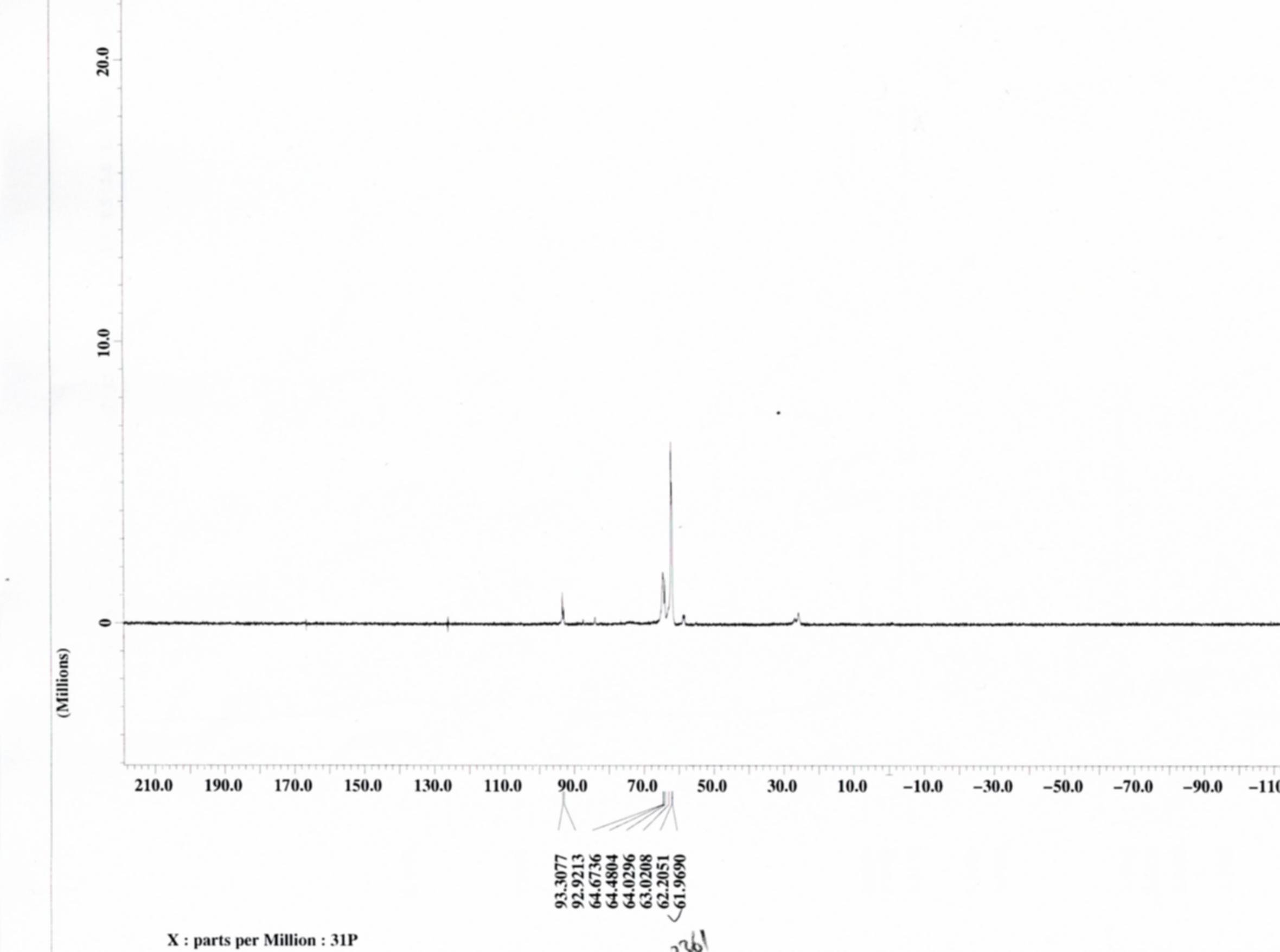


Fig. 12. ^31^P NMR spectra of the compound 3


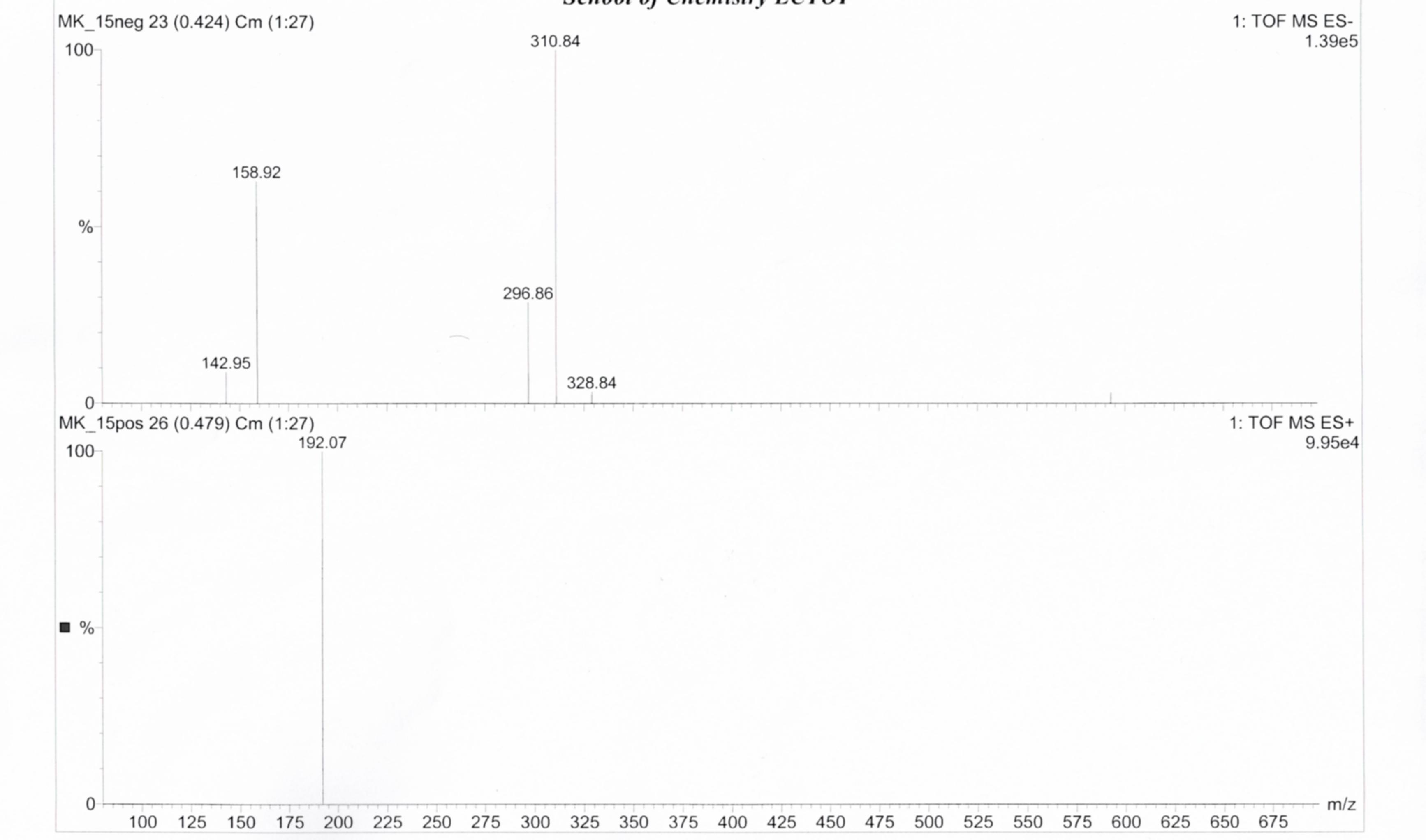


Fig. 13. Mass spectra of the compound 3

**[Cu{Fe(η_5_-C_5_H_5_)(η_5_-C_5_H_4_P(OR)S_2_)(PPh_3_)_2_}] (R = myrtanyl) (1a)**

Fig.14. IR spectra of the compound 1a


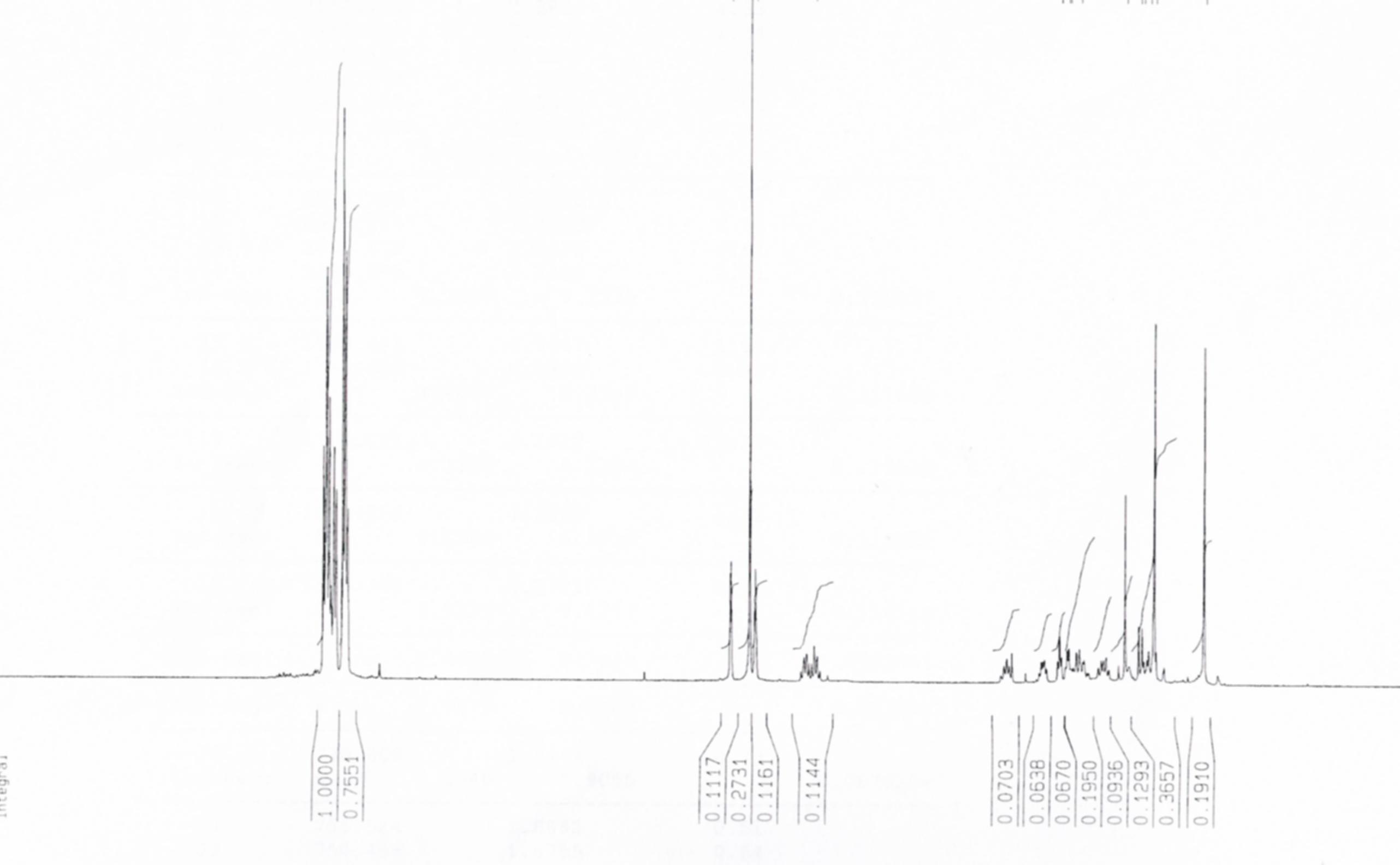


Fig.15. ^1^H-NMR spectra of the compound 1a

**
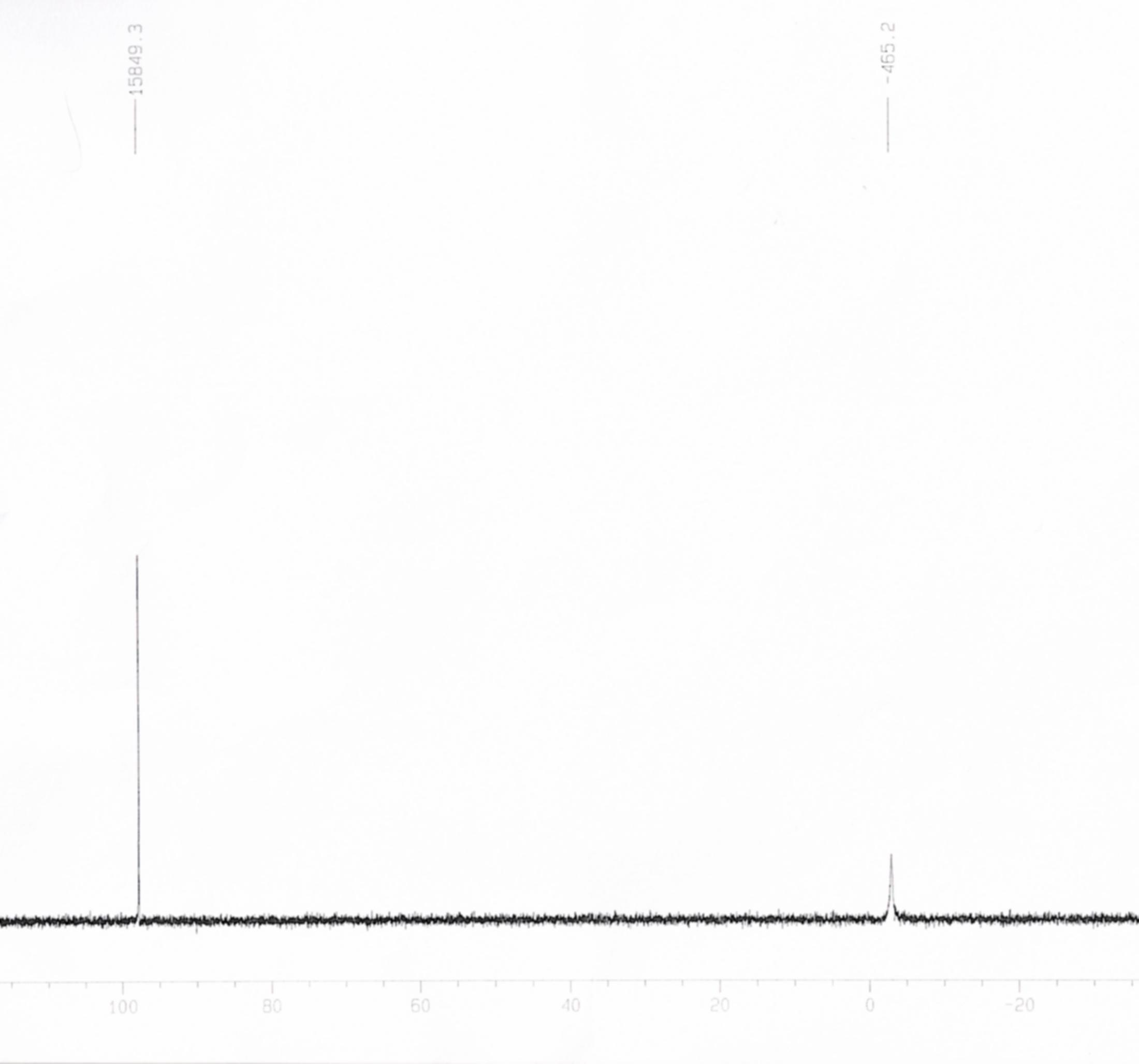
**

Fig. 16. ^31^P NMR spectra of the compound 1a

**[Ag{Fe(η_5_-C_5_H_5_)(η_5_-C_5_H_4_P(OR)S_2_)(PPh_3_)_2_}]_2_ (R = CH_3_) (4)**


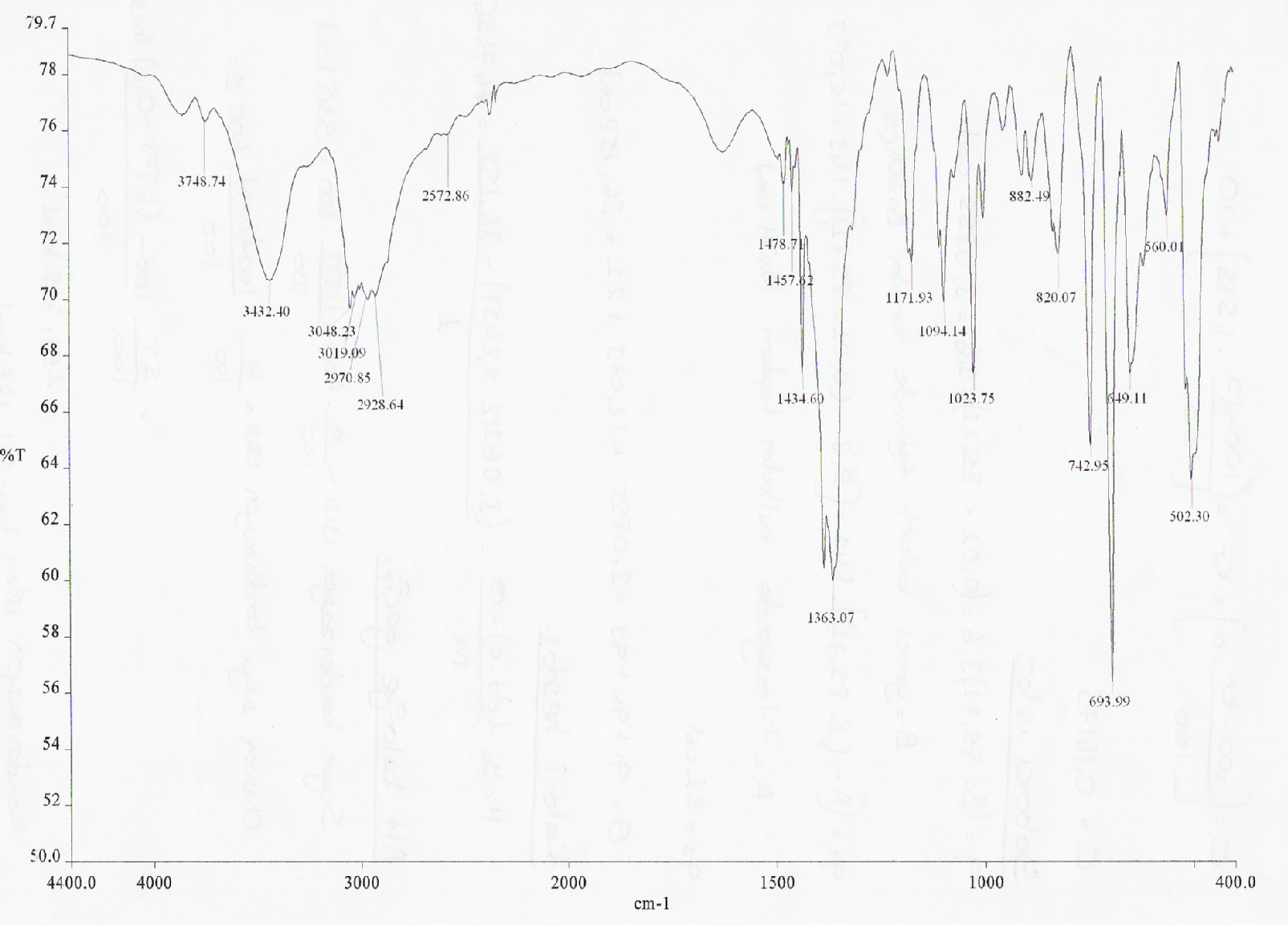


Fig.17. IR spectra of the compound 4


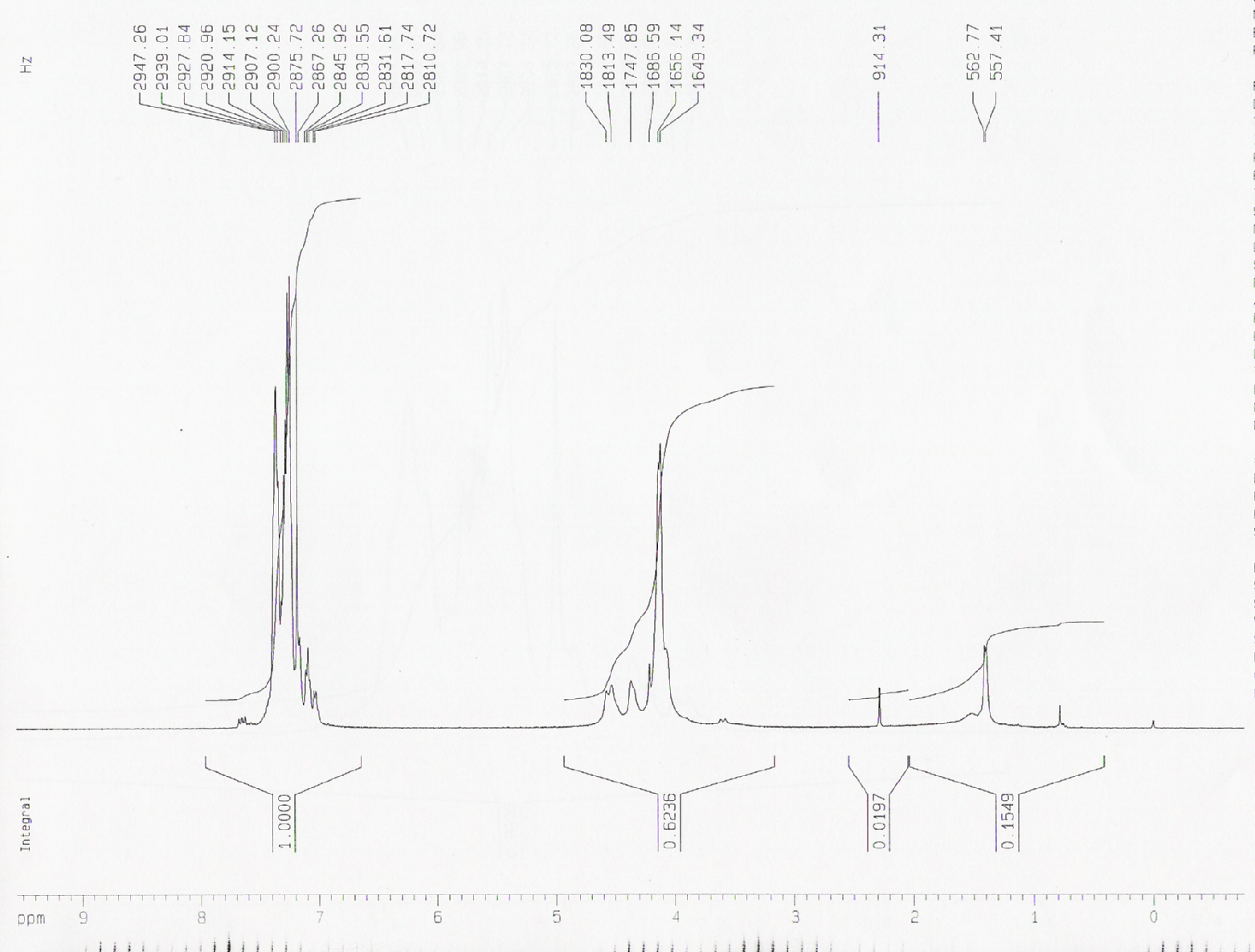


Fig.18. ^1^H-NMR spectra of the compound 4


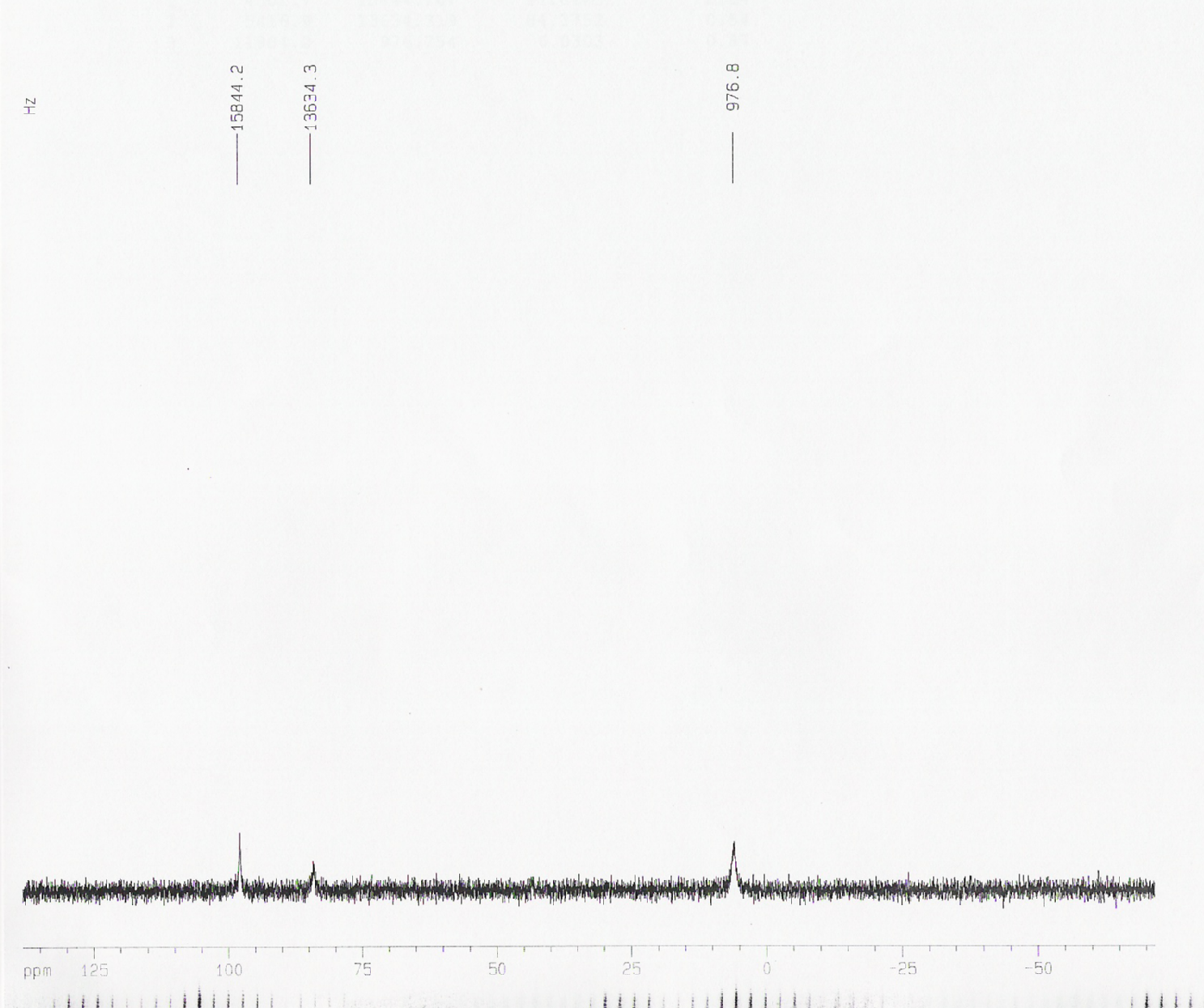


Fig. 19. ^31^P NMR spectra of the compound 4


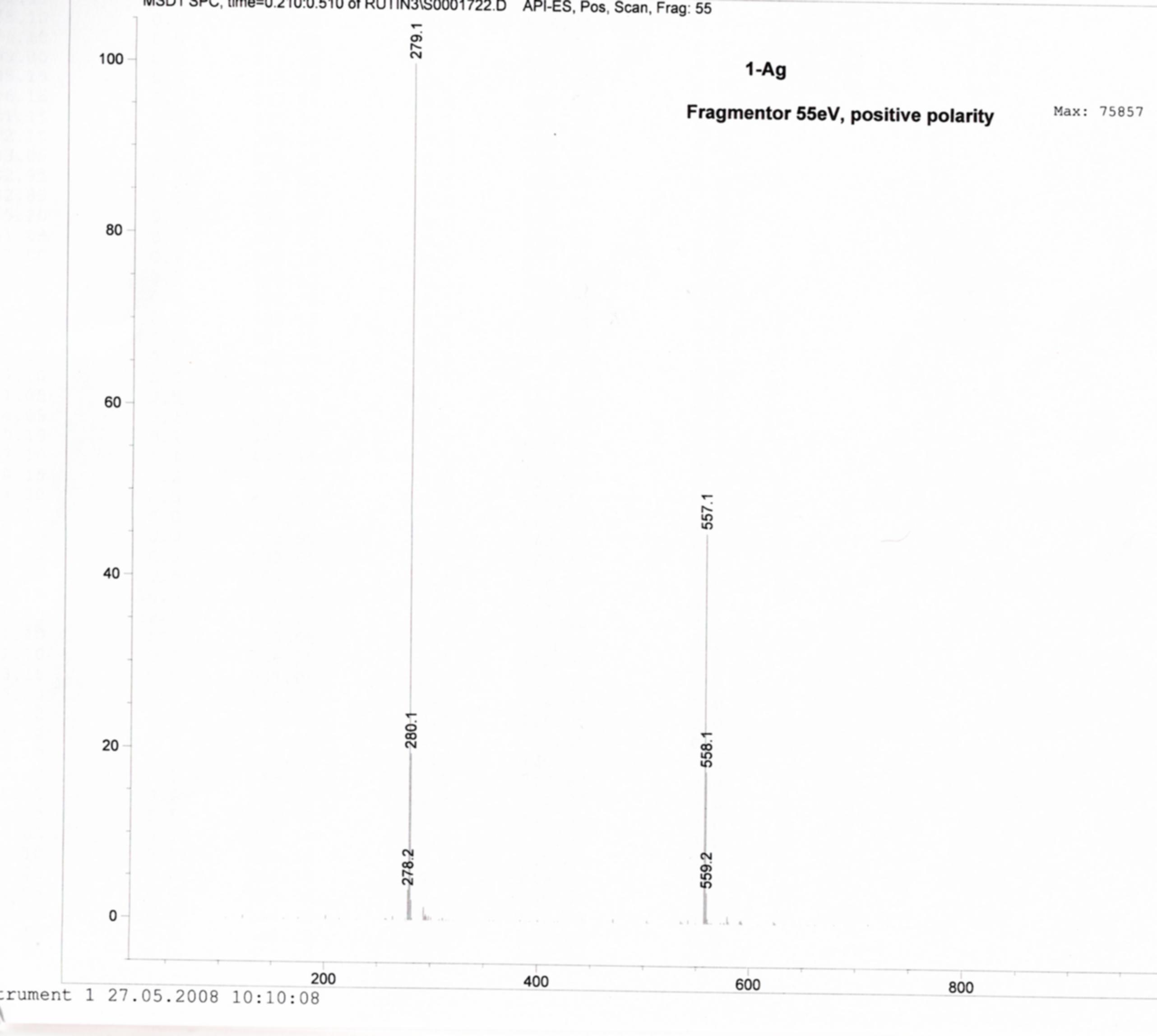


Fig. 20. Mass spectra of the compound 4
